# Engagement with video content in the blended classroom

**DOI:** 10.1042/EBC20210055

**Published:** 2022-04-29

**Authors:** David P. Smith, Nigel J. Francis

**Affiliations:** 1 Department of Biosciences and Chemistry, Sheffield Hallam University, Sheffield SW1 1WB, U.K.; 2 School of Biosciences, Cardiff University, The Sir Martin Evans Building, Museum Avenue, Cardiff CF10 3AX, U.K.

**Keywords:** Engagment, Interaction, learning, Screencast, Video

## Abstract

Blended learning is becoming the expected norm for core content delivery in many institutions. Pre-recorded videos in the form of screencasts are the primary delivery method, with students being asked to engage with the content in this medium. Usage is only likely to increase into the future as delivery moves away from traditional lectures and seminars. In this perspective, we look at the use of video material as a means of content delivery and how to help students engage with it. Theoretical literature around cognitive loading and active learning, alongside personal experience of delivery, is drawn on to give a framework for creating engaging recordings and learning activities.

Within this perspective, we set out to share our practice and understanding around the use of videos as a teaching tool to deliver content, prepare for practical experiences, deliver assessment, and give feedback to both large groups and individuals. Our approaches are based on literature informed evidence and our suggestions on personal observations, research [1] and the collected experience of personal learning networks (#DryLabsRealScience). We draw on our personal, lived experience as educators and lifelong learners, who as a dyslexic benefit from working with non-text-based media, and as a partially deaf individual, who values being able to playback recordings and listen carefully to classroom activities. The authors have both been awarded the Royal Society of Biology Higher Education Bioscience Teacher of the Year award with case studies centred on the development and dissemination of student interaction in the physical (Smith 2019), and digital spaces (Francis 2021).

Student engagement is a term that has many and diverse meanings [2]; here, we use the term in relation to interaction and engagement with video content leading to improved learning outcomes and understanding [3]. Learners’ engagement with video content often extends to individual motivation to progress in their education. When students display high levels of engagement in developing their understanding, they are more likely to excel academically, feel a sense of connection with their university, and have a more positive social–emotional well-being [4]. In higher education, students are self-directed learners, and the way academics interact with their cohorts significantly affects the students’ motivation to study [5], and this includes the recorded material presented to them. Within the biosciences, we have observed that as a result of the COVID-19 pandemic, the primarily mode of delivery has shifted from lecture, or seminar-based to online teaching formats, often providing content as pre-recorded videos [6]. Laboratory teaching has also shifted to include pre- and post-learning activities, often delivered by pre-recorded or streamed video content [7]. With blended learning becoming the expected norm [6,8,9], video usage is only likely to increase. In this perspective, we look at the use of video material as a means of content delivery and how to help students engage with it and develop their understanding.

## Video as a learning tool

No one single definition fully captures hybrid or blended learning, but it is generally accepted as a combination of face-to-face and online teaching integrated into one cohesive experience [10]. Within this perspective, blended learning is set within the context of the bioscience as an approach by which complex threshold concepts are delivered through digital videos distributed through a virtual learning environment. Well-designed videos both support and supplement learning, bridging the digital and physical spaces and challenging the dominance of text-based materials [11]. They act as a precursor to active learning within the classroom [12] or laboratory environment [13–15], as an additional means of consolidating prior learning [16], a supplement to taught material [17], and a means of personalising assessment and feedback [18,19]. A recent study by Mustafa et al. (2021), investigating the use of videos to teach anatomical dissections, highlighted students favour a blended approach and would not want videos to replace face-to-face instruction [20]. The potential of blended learning as a flexible, accessible, and digitally enabled model has created a wave of activity amongst higher education institutions (HEIs), resulting in a broad range of practices.

The use of videos as a learning tool does have a sound theoretical basis. In 1969, Paivio presented his dual coding theory, proposing that our brains have distinct but linked regions for processing visual and auditory information [21]. Dual coding suggests that the maximal cognitive learning benefits occur when complementary information is presented simultaneously to both systems, as occurs in well-designed videos and teaching sessions [21,22]. As well as potentially helping students learn the material more efficiently, video lectures offer additional benefits to students, which centre around control over their learning. The ability to pause and rewind videos, unlike live sessions, allows students to manage their cognitive load, specifically the intrinsic load of the task [23], which is defined as the load placed on the working memory inherent to the task itself [24]. There are several alternative benefits to the use of videos as the primary means of core content delivery; commuter students, those with part-time work or caring responsibilities unable to attend a live session, can still be part of the learning experience. The ability to work at one’s own pace and review material later helps develop self-directed learning strategies. Well-structured videos also allow for a reduction in extraneous load [25], which is where the working memory becomes ‘distracted’ by content that is not inherent to the learning outcome [24]. Through editing, academic staff can limit these extraneous details that could otherwise overload the working memory capacity and reduce the ability of the students to remember new material [24]. Brame (2016) provides an in-depth look at effective ways of managing cognitive load and maximising video effectiveness as an instructional tool [26].

## Engaging in recorded materials

Although the material can be presented in any format, it is commonplace for the core content to be in the form of a video and in many institutions, these have taken the place of large group teaching lectures. There are areas of caution, however, as distant learning environments have been linked to waning subject interest and motivation among students due to feelings of isolation online [27]. Traditional video lectures can be a very linear learning experience, and many students will not be actively engaged whilst watching a video recording, with the level of student learning being proportional to the degree of interactivity in the resource [28]. Interactivity is more easily facilitated in environments where students can work together in small groups, but asynchronous videos are different. To gain engagement, students must want to watch the videos; linking to their course and the application of the core content is critical. In our experience of using videos in teaching:

- **Audio is key:** The first thing students will notice about your screencast video is not the video itself but how clear the audio is. Investing in a good quality microphone will ensure your audio is not muffled or unclear.
- **Planning:** Creating an outline for your video, especially for instructional material, enables you to organise your thoughts and design a ‘roadmap’ for your screencast. Students will quickly lose interest if the content jumps back and forth between thoughts.
- Open your screencast by specifying the learning objectives, then deliver your content and give a summary. Recap your key points and tell your viewers what they have learned.
- **Video length:** Duration is negatively correlated with engagement. There is a reason TikTok is addictive [29]. The optimum length has been quoted at approximately 10–20 min for educational material [25,30]. If you have a lot of content, consider breaking it up into separate, more focused segments (but do be aware of the total run time). Five 10-min videos are often more effective than one 50-min video [25]. This chunking also has the advantage that when the time comes to replace or re-record the video, it can be done in sections.
- **Production:** There is no need for a video to be highly produced. Students currently do not expect Hollywood levels of production. Evidence in the literature suggests that the rougher nature of screencasts can make them feel like a one-on-one tutorial, increasing connection with the student [25]. Eight common styles of video have been identified as frequently used in HE – see Choe et al. (2019) for a more in-depth discussion about the pros and cons of each and their impact on learner outcome [31].
- **Accessibility:** It is crucial to consider accessibility and provide captions or transcripts so you can support students who might struggle with accessing videos; indeed, this is a legal requirement for HEIs in the United Kingdom under the Public Sector Bodies (Websites and Mobile Applications) Accessibility Regulations 2018. Alongside the screencast, PowerPoint slides or notes that replicate the information can also be provided, presenting the material in a range of formats. Be considerate of the amount of text on the screen; many students engage via mobile devices where text-heavy slides cannot be easily read.

## Scaffolding the learning

The act of watching a video is a passive experience [28], and watching does not mean learning has occurred, in the same manner, that passively listening to lectures does not mean one understands the content. Engagement can come through active learning, which is an instructional strategy that encourages students to participate in the learning process rather than passively receiving instruction [5,32,33] and can be as simple as embedding concept checking questions in the video to encourage cognitive activity [26,34]. Active learning is student-centred and requires individuals to take ownership of their own learning experiences [35], with numerous studies demonstrating the benefits [36]. These range from reduced failure rates [37], grade improvement [38], and increased knowledge retention [39]. In relation to video delivery, several strategies have been employed to increase engagement [40].

- **Release in stages:** Releasing all the content at once to students can be overwhelming. An alternative strategy is to release content on a week-by-week basis with clear instructions on what to do at each stage with links to supporting materials.
- **Workbooks:** In the traditional lecture, active learning components would have been added in, such as quizzes to test understanding and tasks or activities to consolidate learning [17,41–43]. Physical or digital workbooks can be produced that give the students tasks to do while watching the videos that are centred on the key learning points. Here short, chunked videos can be utilised with each page or section of a workbook being linked to a video. Software such as Panopto or Google forms allows self-marking questions to be embedded, giving instant student feedback and the tutor the option of monitoring engagement and understanding [44].
- **Assessment-linked consolidation tasks:** A consolidation task takes the learning of the videos and asks the students to engage with it through analysis, evaluation and creativity. Like it or not, assessment can be a significant driver for engagement. Consolidation tasks that prepare the students for later assessment give a reason to engage in the material. These tasks can be problem-based, analysis of information/data or simply a direction to the key learning points to consolidate and apply.
- **Asynchronous support:** With students accessing videos at a time of their choosing, tutor support can become asynchronous and having clear lines of communication with the tutor is key. This can be achieved via discussion boards, tools like Padlet, e-mail communications and *ad-hoc* drop-in sessions.

## Classroom preparation and consolidation

Given core knowledge is held and delivered in the digital realm, in-class time can be dedicated to exploring topics in greater depth and creating meaningful learning [5,32,33]. One of the most well-utilised forms of active learning is flipped learning, in which the traditional view of lecture or seminar learning is inverted [12,45]. In this model, students are introduced to the learning material, often through video prior to a session, with face-to-face time being used to conduct problem-solving activities designed to deepen understanding through discussion and application [1,45–47] and has been shown to increase critical thinking skills [48]. Many students have reported to the authors, however, that the volume of material to be watched prior to a session(s) can be overwhelming due to cognitive overloading, and care should be taken to manage this cognitive load [23]. These personal observations are echoed in the literature, with students reporting that they would rather not attend than arrive having not engaged or do not find videos an effective way to learn [49]. Nevertheless, flipped learning is an effective tool, and a recent study by Fakhoury et al. (2021) demonstrated that students seem to prefer the flipped approach to learning [50]. As an alternative in-class time can be spent on the core learning points with the video material to be watched later, adding depth and breadth. In-class activities are then open to the full range of active learning methods [33]. Peer interaction and individual attention here is key; the aim of these sessions is not to repeat the learning in the videos, instead allow the knowledge to be introduced, used, or applied and to build social interactions between students.

Effective active learning tasks are linked to the real-world application of the material and, if needed, to the summative assessment tasks. Consolidation activities from the workbooks perform well here as a focus to explore and apply learning. Student response systems allow the tutor to probe the level of knowledge, revealing any misunderstandings which can then be addressed in real-time [42,51,52]. Collaborative working through shared documents gives a focus to the session and can also be used in the hybrid environment with remote and physically located students working together [53]. The tutor conducts sense checking of this work to correct any misconceptions. One clear benefit of online live delivery is the use of the chat function, with back-channel conversations increasing popular during physical and digital delivery. What is key is for students is the option to use text response systems in class as a means of asking questions either openly or anonymously [54].

## The blended lab

Blended learning and the use of recorded material can equally be expanded to the practical delivery of the laboratory experience [13–15]. Many of the lessons learned around effective engagement in taught sessions hold true for the practical experience. In the same way that recorded material can be used to prepare students for active learning, videos have been shown to be a highly effective tool to introduce students to key laboratory concepts, including health and safety and the use of equipment. These videos are often used as a means of introducing students to laboratory equipment and their practical use and are embedded into pre-sessional materials [13,14]. Using tools like EdPuzzle allows for interactivity to be embedded in these resources with real-time feedback to students [55]. Simulations can generate unique datasets allowing the students to problem solve quickly in a consequence-free environment prior to the laboratory session [56]. A wide range of innovations has been shared through the #DryLabsRealScience network and materials made available on the lectureremotly.com website. What is clear is that blended learning models extend to the point of student preparation and support for the laboratory; what cannot be replaced digitally, however, is the psychomotor skills that are developed through the physical act of laboratory work [57].

## Ending thoughts

The use of video as a primary means of delivering content, explanation or consolidation is now firmly embedded in teaching practices. Although the technology around recording and delivery is likely to improve and adapt with time, the fundamental medium as a means of communication will remain. It is up to us as educators how we use this medium to engage and teach our students.

## Open Access

Open access for this article was enabled by the participation of Cardiff University in an all-inclusive *Read & Publish* agreement with Portland Press and the Biochemical Society under a transformative agreement with *JISC*.

## Abbreviation

HEI: higher education institution
HE: higher education
